# Concurrent SPECT/PET-CT imaging as a method for tracking adoptively transferred T-cells in vivo

**DOI:** 10.1186/s40425-016-0131-3

**Published:** 2016-05-17

**Authors:** Sasha E. Stanton, Janet F. Eary, Edmond A. Marzbani, David Mankoff, Lupe G. Salazar, Doreen Higgins, Jennifer Childs, Jessica Reichow, Yushe Dang, Mary L. Disis

**Affiliations:** Tumor Vaccine Group, Center for Translational Medicine in Women’s Health, University of Washington, Seattle, WA 98109 USA; Department of Radiology, University of Alabama at Birmingham, Birmingham, AL 35249 USA; Department of Radiology, University of Pennsylvania, Philadelphia, PA 19104 USA

**Keywords:** Adoptive T-cell therapy, HER2, breast cancer, Indium-111 labeled, FDG, PET-CT, SPECT

## Abstract

**Background:**

The ability of T-cells to traffic to and penetrate tumors impacts the clinical efficacy of T-cell therapy therefore methods to track transferred T-cells in vivo are needed. In this preliminary report, we evaluated the use of concurrent SPECT/PET-CT imaging to monitor the egress of HER-2/neu specific T-cells in a breast cancer patient with extensive bone-only metastatic disease.

**Findings:**

Indium (In-111) labeled T-cells demonstrated similar or greater viability than unlabeled T-cells at either a low or high dose of In-111 over a 24-h incubation period in vitro. The function of labeled or unlabeled T-cells was not significantly different (*p* > 0.05) at either dose. T-cells trafficked to all sites of metastatic disease and infiltrated the tumor as assessed by SPECT imaging. In-111 uptake at 24 h after infusion varied from 3.8 (right proximal humerus) to 6.3 (right sacrum) background corrected counts per pixel and remained elevated at 48 h. Concurrent PET-CT imaging demonstrated a fluorodeoxyglucose flare, measured by increase in tumor site uptake as high as 32 % and at most sites of disease at 48 h. This flare was associated with focal pain after T-cell infusion at metastatic sites. The patient had stable disease for 18 months after completion of T-cell therapy.

**Conclusion:**

Concurrent SPECT/PET-CT imaging, over a 48-h period after T-cell infusion, provided evidence of T-cell homing to all disease sites as well as a tumor metabolism flare response. This technique may be useful for monitoring T-cell trafficking after autologous as well as chimeric antigen receptor T-cell infusion.

**Trial Registraion:**

Trial registered at ClinicalTrials.gov registration number NCT00791037, registered 13 November 2008.

## Background

Adoptive T-cell therapy using chimeric antigen receptor, tumor infiltrating, or antigen specific T-cells shows significant clinical responses in hematologic cancers but few durable responses in solid tumors [1–3].Limitations include the inability of antigen specific T-cells to traffic to and penetrate solid tumors and significant variations in tumor T-cell infiltration [4]. For example, in adoptive transfer of HER2 specific T-cells, indium 111 (In-111) labeled cells could traffic to the bone marrow but not to liver metastases [5]. Methods are needed to monitor infused T-cells in vivo, evaluate their function, and demonstrate whether T-cells penetrate all metastatic sites.

Readily accessible approaches for monitoring T cells in vivo are tracking T-cells labeled with In-111 by single photon emission computed tomography (SPECT) imaging or evaluating T-cell activity by fluorodeoxyglucose positron emission tomography (PET-CT) [6]. As part of a clinical trial, we piloted the concurrent use of SPECT and PET-CT imaging to assess the homing capability and inflammatory function of adoptively transferred antigen specific T-cells at various metastatic sites.

## Findings

### Indium-111 labeling does not impact T-cell viability nor diminish antigen specific cytokine secretory function

**Fig. 1:**
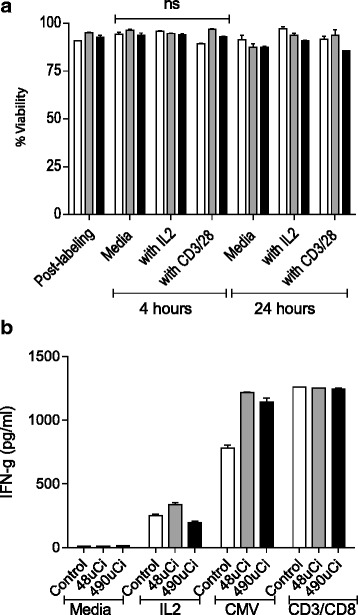
Indium-111 labeling does not impact T-cell viability nor diminish antigen specific cytokine secretory function. **a** Cell viability of unlabeled controls (white), 48uCi (grey), and 490uCi labeled T-cells (black) in media only, with IL-2, or CD3/CD28 bead stimulation after culture for 4 and 24 h. **b** Levels of IFN-g (pg/ml) secreted in 48 h culture supernatants of unlabeled controls (white), 48uCi (grey), and 490uCi (black) labeled T-cells cultured with media only, IL-2, after CMV antigen stimulation, or CD3/CD28 stimulation. Columns and bars represent the mean of duplicates (± SE) for each condition for both donors

T-cells labeled with 49uCi and 480uCi demonstrated similar viability as compared with unlabeled cells at 24 h when plated at comparable cell concentrations (Fig. 1a). After IL-2 stimulation, 49uCi labeled T-cells were 93.7 ± 1.1 % viable (*p* = 0.16) and 480uCi labeled T-cells were 90.9 ± 0.3 % viable (*p* = 0.03) as compared to unlabeled T-cells. After CD3/CD8 stimulation, 49uCi labeled T-cells were 93.9 ± 2.7 % viable (*p* = 0.537) and 480uCi labeled T-cells were 85.7 ± 0.0 % viable (*p* = 0.08) as compared to unlabeled T-cells. At 4 h there were no statistically significant differences between groups (Fig. 1a).

In-111 labeling did not diminish the ability of T-cells to secrete IFN-gamma (IFN-g). When cultured with IL-2, 49uCi labeled T-cells secreted 336.0 ± 25.4 pg/mL (*p* = 0.09) and 480uCi labeled T-cells 194.5 ± 16.3 pg/mL (*p* = 0.07) IFN-g as compared to unlabeled T-cells. 49uCi labeled T-cells 1215.0 ± 8.5 pg/mL (*p* = 0.03) and 480uCi labeled T-cells 1139.0 ± 46.7 pg/mL (*p* = 0.02) secreted more IFN-g in response to CMV antigen than unlabeled cells. All groups responded equally to stimulation with CD3/CD28 beads (*p* < 0.05 for all groups) (Fig. 1b).

### HER2 specific T-cells trafficked to and infiltrated all sites of metastatic disease by 48 h

**Fig. 2:**
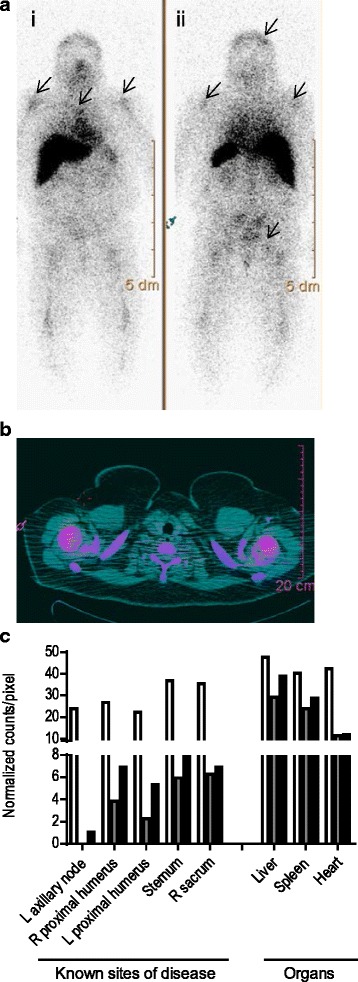
HER2 specific T-cells trafficked to and infiltrated all sites of metastatic disease over a 48 h period. **a** Whole body planar images of In-111 labeled T-cells (i) anterior and (ii) posterior views 24 h after infusion show focal areas of increased uptake in the left postero-lateral skull, both humeral heads, mid-sternum, and R sacrum (arrows). **b** SPECT/CT fused image of In-111 T-cells acquired 24 h post infusion; humeral heads show T-cell infiltrates (pink). **c** In-111 uptake in counts/pixel normalized to background at 4 h (white), 24 h (grey), and 48 h (black) after infusion

The patient had known metastases to her skull, sternum, bilateral proximal humeri, and right sacrum. Labeled HER2 specific T-cells trafficked to all metastatic sites of disease by 24 h (Fig. 2a). Figure 2b shows a representative example of labeled T-cell infiltration into both humeral metastases. At 24 h the corrected In-111 uptake varied from the left proximal humerus at 2.3 corrected counts per pixel (ccpp) to the right sacrum at 6.3 ccpp. By 48 h, the corrected In-111 uptake had increased by 44 % in the right proximal humerus, 56 % in the left proximal humerus, 28 % in the sternum, and 9 % in the right sacrum. Similar to other studies, In-111 uptake was present in the spleen, liver, and heart at 4 h and remained in these organs at 48 h (Fig. 2c) [7, 8].

### FDG PET-CT demonstrated acute increases in tumor metabolism at most metastatic sites 48 h after T-cell infusion

**Fig. 3:**
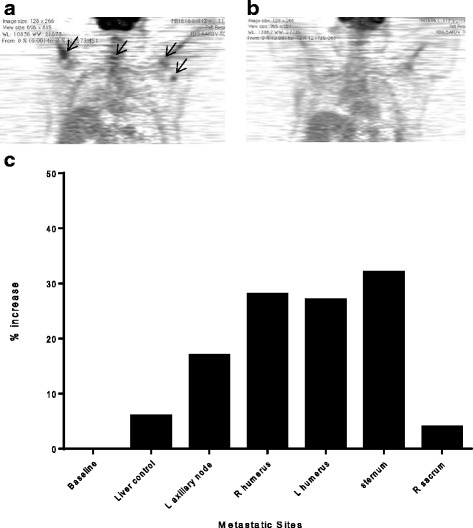
FDG PET-CT demonstrates acute increases in SUV at most metastatic sites over a 48 h period after T-cell infusion. **a** FDG PET-CT image 48 h post T-cell infusion. **b** FDG PET-CT image 3 months following T-cell infusion. **c** FDG uptake as % increase over baseline 48 h

FDG PET-CT imaging demonstrated increased FDG uptake at most sites of metastatic disease by 48 h suggesting the trafficking HER2 specific T-cells were functional and activated (Fig. 3a). There was between 17 % (left axillary lymph node) and 32 % (sternum) increase compared to baseline scans (Fig. 3c). These increases in standardized uptake value (SUV) in metastases are higher than the 6 % increased physiologic uptake in the liver (Fig. 3c). Furthermore, SUV uptake in the left axillary lymph node was higher than soft tissue background both at baseline (ratio of lymph node metastasis to soft tissue background is 2.0) and at 48 h (ratio of 2.69) and the SUV uptake in the humeral bone metastases were higher than background bone marrow uptake both at baseline (ratio of left humoral metastasis to left bone marrow background is 1.44) and at 48 h (ratio of left humoral metastasis to left bone marrow background is 1.8). The right sacrum did not have an increase in uptake at 48 h, with FDG SUV lower than liver control. The increased FDG uptake at sites of metastatic disease was not evident 3 months after infusion (Fig. 3b).

Location of FDG tumor uptake mirrored the patient’s report of grade 1 or 2 bone pain flares localized to sites of metastatic disease experienced with successive T-cell infusions. After completion of T-cell infusions, the patient had similar boney pain during HER2 specific booster vaccinations. On completion of the study, the patient experienced stable disease without changes in therapy for 18 months.

## Conclusion

The use of SPECT imaging is an effective modality for tracking T-cells in vivo without impacting their function. Early trials have demonstrated that Melan-A-specific cytotoxic T-cells labeled with In-111 can traffic to sites of metastasis by 48 h and remain up to 14 days [8]. We demonstrate T-cells homing to multiple sites of breast cancer deposits through In-111 labeling.

Changes in FDG tumor uptake quantitated by PET-CT are a functional measure usually associated with disease progression. In 28 breast cancer patients with bone dominant disease, higher tumor SUV predicted a shorter time to disease progression (*p* < 0.006) [9]. In our patient, an acute increase in tumor FDG uptake was associated with disease stabilization. After adoptive T-cell therapy for nasopharyngeal carcinoma or breast cancer, pain at sites of disease temporally related to T-cell infusion correlated with clinical response in specific lesions [3, 10]. Acute increases in tumor metabolism by FDG PET-CT allows quantitation of inflammation induced by activated T-cells and may provide a measure of the level needed for disease regression. More data is needed to calculate a correlation of clinical response with specific changes in SUV.

Optimizing adoptive T-cell therapy for all patients requires an understanding of the trafficking and function of these cells in vivo. This pilot data demonstrates that concurrent In-111 T-cell SPECT and FDG PET-CT imaging, modalities available at any US medical center, can evaluate T-cell homing, tumor penetration by T-cells, and T-cell induced inflammation. These preliminary findings will be expanded in further trials.

## Materials and methods

### T-cell viability and cytokine secretion after In-111 labeling

Peripheral blood mononuclear cells (PBMC) were obtained by apheresis from CMV positive donors. T-cells were cultured with CD3/CD28 beads (Life Technologies), diluted 0.6-1x10^6^/ml, and cultured with IL-2 (30U/ml) every 3 days. Day 12, CD3/CD28 beads were removed and expanded T-cells, >90 % CD3^+^, were labelled with 48uCi or 490uCi In-111 at GE Healthcare (Seattle, WA). 2 mCi In-111 oxine (Covidien) was added to 200x10^6^ cultured T-cells for 30 min and resuspended in media with non-labelled cells as controls. Cells (1x10^6^/ml per group) were cultured at 37 °C with (1) media only, (2) IL-2 (30U/ml), (3) CD3/CD28 beads (2:1 ratio), or (4) CMV lysate (2.5ug/ml) with irradiated autologous PBMC. 100ul cell suspension was removed at 4 and 24 h. Numbers of live and dead cells were counted in duplicate using trypan blue exclusion and percent viability calculated. Supernatants were collected from the cultured cells 48 h after stimulation and IFN-g release was measured using ELISA (eBioscience).

### Clinical protocol

After informed consent, the patient was enrolled in IRB approved trial NCT00791037. Imaging protocols were approved by IRB and radiation safety. Enrollment criteria included (1) metastatic treatment refractory HER2+ cancer, (2) lesions quantifiable by imaging, and (3) no cytotoxic chemotherapy for 1 month before the first T-cell infusion. The patient was vaccinated with three HER2 specific peptide-based vaccines weekly for 3 weeks [11]. Two weeks after the last vaccine, PBMC were collected by leukapheresis and expanded *ex vivo* as previously described [3]. Cells were administered as a dose escalation with 5x10^9^ (dose 1), 5x10^10^ (dose 2), and 5x10^11^ (dose 3) total cells.

### Indium labeling and SPECT imaging

1 mCi In-111 oxine (Covidien) was added to 10x10^6^ HER2 antigen cultured T-cells for 30 min at room temperature and resuspended in normal saline (GE Healthcare Nuclear Pharmacy). Labeled cells were infused prior to the third T-cell infusion. Migration of T-cells was evaluated at 4, 24 and 48 h by whole body planar and SPECT scintigraphy gamma camera imaging (Phillips Precedence; GE Medical Systems) equipped with medium energy collimators. In-111 lesion uptake was quantified by region of interest (ROI) in each metastasis with background subtracted from adjacent soft tissues in counts/pixel. At each time point an aliquot of the injectate was also imaged to calculate radioactive decay for image analysis. Data is reported as counts/pixel normalized to background (ccpp).

### PET-CT imaging

The patient was injected with an activity of 310 MBq (10 mCi) of fluorodeoxyglucose (FDG). Imaging was performed by PET-CT scanner (Discovery LS; GE Medical Systems) in high sensitivity mode and consisted of a torso survey covering 5 adjacent 15-cm axial fields. Baseline FDG PET-CT scan was performed in parallel with SPECT imaging prior to infusion and at 48 h. FDG PET-CT was also performed 3 months after the last T-cell infusion. The FDG uptake in metastases was measured as the tumor ROI compared to an equivalent background normalized by injected dose (SUV). Change in FDG uptake early after T-cell infusion was reported as the ratio of FDG SUV per metastatic site increase compared to baseline.

### Ethics Approval and Consent to Participate/Publish

The human subject research included in this manuscript was performed in accordance with the declaration of Helsinki and the IRB of record for this study is Fred Hutchinson Cancer Research Center/University of Washington Consortium IRB. This review includes a Scientific Review Committee and IRB review of original application, modifications and annual renewals. IRB Reference Number: IR# 6658. Written consent included consent for publication.

## Abbreviations

SPECT: single photon emission computed tomography
PET-CT: fluorodeoxyglucose positron emission tomography
FDG: fluorodeoxyglucose
In-111: indium 111
IFN-g: interferon-gamma
SUV: standardized uptake value
Ccpp: corrected counts per pixel
